# The relationship between obstructive sleep apnoea and erectile dysfunction: An underdiagnosed link? A prospective cross‐sectional study

**DOI:** 10.1111/and.14504

**Published:** 2022-07-11

**Authors:** Elena Cantone, Matteo Massanova, Felice Crocetto, Biagio Barone, Fabio Esposito, Davide Arcaniolo, Fabrizio Corlianò, Luigi Romano, Gaetano Motta, Antonio Celia

**Affiliations:** ^1^ Department of Neuroscience, Reproductive Sciences and Dentistry ‐ ENT Section University “Federico II”, AOU “Federico II” Naples Italy; ^2^ Head and Neck Department UOC Otorhinolaryngology, AOU “Federico II” Naples Italy; ^3^ Department of Urology Southend University Hospital NHS Foundation Trust Southend‐On‐Sea UK; ^4^ Department of Neurosciences, Reproductive Sciences and Odontostomatology University of Naples "Federico II" Naples Italy; ^5^ Department of Woman, Child and General and Specialized Surgery, Urology Unit University of Campania "Luigi Vanvitelli," Naples Italy; ^6^ Department of ENT San Bassiano Hospital Vicenza Italy; ^7^ Department of Mental and Physical Health and Preventive Medicine, Head and Neck Surgery Unit University of Campania "Luigi Vanvitelli," Naples Italy; ^8^ Department of Urology San Bassiano Hospital Vicenza Italy

**Keywords:** ED, Erectile dysfunction, Obstructive sleep apnoea, OSA, SatO2

## Abstract

This cross‐sectional study aimed to investigate the prevalence and clinical characteristics of erectile dysfunction in patients with obstructive sleep apnoea. We enrolled 133 male patients with suspected obstructive sleep apnoea. Ear, nose and throat evaluation, laboratory tests, body mass index, Epworth sleepiness scale, 5‐international index of erectile function, overnight ambulatory polygraphy and drug‐induced sleep endoscopy patterns were assessed. Eighty patients reported obstructive sleep apnoea. 60% (*n* = 48) reported erectile dysfunction. Statistically significant correlations were found between 5‐International Index of Erectile Function and age, hypertension, diabetes, Epworth sleepiness scale, apnoea‐hypopnea index score, O_2_ saturation‐nadir, and oxygen desaturation index. Age, diabetes and O_2_ saturation‐nadir were independent predictors of erectile function. Epworth sleepiness scale, apnoea‐hypopnea index score, O_2_ saturation‐nadir, oxygen desaturation index and albumin were higher compared to patients without erectile dysfunction. No statistically significant differences were reported for drug‐induced sleep endoscopy patterns and erectile dysfunction. Patients with obstructive sleep apnoea were at significant risk of having erectile dysfunction. Males with obstructive sleep apnoea should be investigated for erectile dysfunction.

## 1 INTRODUCTION

Obstructive sleep apnoea (OSA) is a condition characterized by repetitive episodes of cessation of breathing during sleep, related to partial or complete obstruction of airways. The estimated prevalence in the general population ranges from 9% to 38%, with over 100 million people affected worldwide (Benjafield et al., 2018; Zhang et al., 2019). OSA, which results in snoring, episodes of hypoxia, disturbed sleep and daytime somnolence, has become a serious health problem with negative effects on the quality of life (İrer et al., 2018; Silva et al., 2016; Stepnowsky et al., 2019). Its risk factors include increasing age, obesity, and male gender (Gabbay & Lavie, 2012; Shazia Jehan et al., 2017; Nigro et al., 2018). Moreover, OSA often coexists with other systemic comorbidities, such as diabetes, hypertension, cardiovascular diseases, and sexual dysfunctions (Bonsignore et al., 2019; Pinto et al., 2016; Zheng et al., 2020). Erectile dysfunction, i.e the inability to achieve or maintain a rigid penile erection suitable for sexual intercourse, affects 52% of men between 40 and 70 years and similarly represents an increasing health concern due to its effects on quality of life and its increasing prevalence, which is estimated to exceed 320 million men worldwide by 2025 (Kessler et al., 2019; Yafi et al., 2016). Among the different causes of ED, such as surgery of the lower urinary tract, prostatic surgery, neurological diseases and metabolic/hormonal disorders, several studies and meta‐analyses confirmed the increased prevalence of ED in patients with OSA (Chen et al., 2015; Crocetto et al., 2021; Kellesarian et al., 2018; Liu et al., 2015; Manfredi et al., 2021; Romero‐Otero et al., 2021; Sperlongano et al., 2014). In addition, the treatment with continuous positive airway pressure (CPAP) can improve erectile function (Li et al., 2010; Pascual et al., 2018). Nevertheless, connections between sleep disorders and sexual problems are largely understudied and OSA syndrome is one of the lesser‐studied risk factors for ED (Kalejaiye et al., 2017). Despite the mechanisms that underlie ED remain unclear, sexual problems are common among men with sleep disorders (Hoyos et al., 2015). So far, the exact pathogenesis linking OSA and ED is not well established albeit it has been postulated the role of shared comorbidities such as diabetes, hypertension and metabolic syndrome in inflammation and vascular impairment present in both diseases (Bouloukaki et al., 2014; Hoyos et al., 2015; Kellesarian et al., 2018). Other possible theories focused on reduced nocturnal erections in the rapid eye movement (REM) periods due to sleep fragmentation, sympathetic hyperactivity after each OSA episode and modifications in the hormonal status of OSA patients (Andersen et al., 2010). Several validated questionnaires as the Berlin questionnaire (BQ), STOP‐Bang questionnaire and the Epworth Sleepiness Scale (ESS), allow non‐specialist clinicians to identify patients at high risk of having OSA (Amra et al., 2018; Pereira et al., 2013). The gold standard for the diagnosis of OSA is polysomnography (PSG) which requires the patient to sleep overnight in a sleep laboratory under observation. As result, it is costly, time‐consuming, often inaccessible and uncomfortable, requiring, in addition, expert technicians (Gregório et al., 2011). For these reasons, most sleep study centres use overnight ambulatory respiratory polygraphy (RP) which is easier and cheaper, being a portable monitoring and recording device, easily usable at home (Berry et al., 2012).

In this study, we aimed to investigate the prevalence and clinical characteristics of ED in patients with confirmed OSA. In addition, we correlated polygraphic and sleep endoscopy parameters (the VOTE—Velum, Oropharinx, Tongue, Epiglottis—classification patterns, that is, a method to classify the site of obstruction in patients with OSA), with erectile function scores, in order to evaluate the potential impact of those parameters.

## 2 METHODS

This prospective cross‐sectional study was approved by the Research Ethics Board of the University of Naples “Federico II” (n. 316/20) and was conducted according to the World Medical Association Declaration of Helsinki Guidelines. All patients gave their written informed consent to the study. From January 2018 to November 2019, we evaluated 133 consecutive male patients complaining snoring, sleepiness, morning dry mouth and tiredness compatible with suspected OSA (Zhang et al., 2019). Exclusion criteria were: age under 18 years; psychiatric, neurological, hepatic, severe cardiac (other than hypertension), pulmonary, oncological and endocrinological (other than diabetes) diseases; previous diagnosis and related treatment for ED. Patients with cardiovascular diseases were excluded from this study due to the well‐known relation with ED (Terentes‐Printzios et al., 2022). Similarly, patients with chronic liver disease were excluded due to the altered hypothalamic–pituitary‐gonadal axis, which could have influenced the erectile function (Burra et al., 2010).

### 2.1 Laboratory data and questionnaires

Full medical history, complete ear nose and throat evaluation and sexual hormonal assessment—serum total testosterone (T), prolactin (PRL), luteinizing hormone (LH), and follicle‐stimulating hormone (FSH) [normal values: T (age 40–59 years) 350–890 ng/dl, (age >60 years) 300–720 ng/dl; PRL 5–15 ng/ml; LH 2–12 and FSH 5–20 mIU/ml]—were obtained from all subjects at the time of the enrolment in the study. C‐reactive protein (CRP), albumin, total cholesterol, low‐density lipoprotein (LDL), high‐density lipoprotein (HDL) cholesterol and triglycerides were assessed as well. The height and weight of all patients were measured, obtaining Body Mass Index (BMI) according to the formula weight (Kg)/height (m)^2^. Patients were subsequently asked to complete the validated Epworth Sleepiness Scale (ESS) and the 5‐International Index of Erectile Function (IIEF‐5) questionnaires. The ESS (range 0–24), is an eight‐item questionnaire used to assess the propensity to fall asleep in various circumstances, with a cut‐off of ≥10 points indicating excessive daytime sleepiness (Trimmel et al., 2018; Vignatelli et al., 2003). The International Index of Erectile Function (IIEF) 5 is an abridged five‐item version of the 15‐item International Index of Erectile Function, and it was developed to assess the erectile function over the previous 4 weeks. A score of ≤ 21 indicates erectile dysfunction (D'Elia et al., 2012; Rosen et al., 1999).

### 2.2 Polygraphy and drug‐induced sleep endoscopy procedure

All subjects underwent overnight ambulatory RP (Weinmann SOMNOlab 2, Hamburg, Germany) according to the American Academy of Sleep Medicine Guidelines (Berry et al., 2012). We recorded the following parameters: electrocardiogram, thoracic and abdominal excursion, oral and nasal airflow by thermistor, breath sounds, body position and oxygen saturation by pulse oximeter. We evaluated Apnea‐Hypopnea Index (AHI), Oxygen Desaturation Index (ODI), total number of events per night, mean arterial blood oxygen saturation (SaO2) and sleep time at SaO2 below 90% (SatO2‐nadir). Apnoea severity was based on the AHI values: mild (AHI 5–15), moderate (AHI 16–30) or severe (AHI >30; Berry et al., 2012). The diagnosis of OSA was made for AHI >5 at RP.

Successively, drug‐induced sleep endoscopy (DISE) was performed, in order to evaluate potential surgical treatment candidates and clinical mismatches.

DISE was performed employing a flexible rhinopharyngolaryngoscope (Storz, Tuttlingen, Germany) in the operating theatre using a propofol target‐controlled infusion (TCI) to achieve a complete evaluation of the upper airways (UA) collapse. Bispectral Index (BIS) was used to check the level of sedation during DISE. Blinded VOTE classification scoring was used for classifying the type of obstruction detected at DISE. The VOTE classification represents a method to assess the type of obstruction, based on the collapse and closure of the airway of different oropharyngeal structures (velum, oropharynx, tongue base, epiglottis; Kezirian et al., 2011).

### 2.3 Statistical analysis

Statistical analysis was conducted using IBM SPSS software (version 25, IBM Corp, Armonk, NY, USA). Descriptive statistics were obtained and reported as means and standard deviations for continuous variables while frequencies and percentages were reported for categorical variables. Normality of data was tested with the Kolmogorov–Smirnov test and parametric tests were used accordingly. Power analysis for sample size assessment was conducted considering, according to current literature, a 50% of prevalence of erectile dysfunction among OSA patients, accordingly to alpha = 0.005 and beta = 0.2 (Feng et al., 2022; Rosner, 2015). Pearson's correlation coefficient was obtained for every variable recorded in relation to IIEF‐5. An independent‐sample t‐test was conducted for continuous variables to compare obtained data between patients with and without ED while the Chi‐Square test was performed, similarly, for categorical variables. A two‐way ANOVA was performed to compare the effect of VOTE classification on the IIEF‐5 score. We further calculated univariate and multivariate linear regression to predict the IIEF‐5 score based on variables that showed statistically significant correlations. *p*‐Value was considered significant for *p* < 0.05.

## 3 RESULTS

**FIGURE 1:**
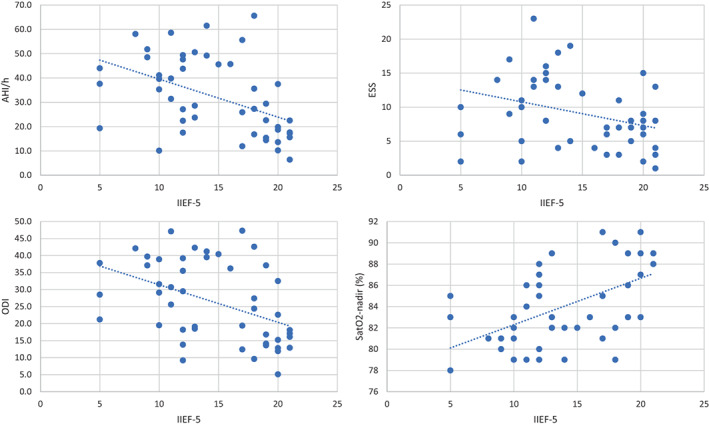
Patients included in the study. COPD, chronic obstructive pulmonary disease; ED, Erectile dysfunction; OSA, obstructive sleep apnoea

According to polygraphic parameters, 80 male patients were diagnosed with OSA, met inclusion criteria and were enrolled in the study (Figure 1). 16 (20%) patients presented a mild OSA (AHI 5–15), 35 (44%) a moderate OSA (AHI 15–30), and 29 (36%) a severe OSA (AHI >30). 60% of patients (48/80), based on IIEF‐5 ≤21 (mean score 18.15 ± 5.63 *SD*), were diagnosed with ED. Laboratory data and RP parameters are reported in Table 1.

**TABLE 1:** Descriptive characteristics

	Mean	*SD*	Range value (min‐max)
Age	54.99	9.2	39 (32–71)
BMI	27.03	3.33	14.9 (20.6–35.5)
ESS	8.13	4.66	22 (1–23)
IIEF‐5	18.15	5.63	20 (5–25)
AHI/h	27.63	15.05	62 (3.6–65.6)
ODI	22.68	11.54	42.7 (4.6–47.3)
SaO_2_‐nadir (%)	85.60	3.86	14 (78–92)
Total Colesterol (mg/ml)	194.75	32.56	168 (104–272)
LDL (mg/ml)	120.66	29.30	141 (38–179)
HDL (mg/ml)	46.71	7.79	46 (33–79)
Triglycerides (mg/dl)	135.78	37.62	147 (57–204)
Albumin (mg/dl)	4.46	0.62	1.9 (3.5–5.4)
Testosterone (ng/dl)	564.26	143.94	509 (351–860)
PRL (ng/ml)	11.68	4.53	16.3 (3.07–19.38)
FSH (mIU/ml)	5.43	2.99	11.5 (0.9–12‐4)
LH (mIU/ml)	6.55	3.09	10.5 (1.5–12)
PCR (mg/L)	3.73	2.32	7.9 (0.1–8)
	Yes	No	
Hypertension (%)	33 (41.3)	47 (58.8)	
Diabetes (%)	12 (15)	68 (85)	
Smoking (%)	21 (26.3)	59 (73.8)	
	Mean	*SD*	
Pack/year	36.95	17.47	58 (9–67)

Abbreviations: AHI, Apnea‐Hypopnea Index; BMI, body mass index; CRP, C‐reactive protein; ESS, Epworth Sleepiness Scale; FSH, follicle‐stimulating hormone; HDL, high density lipoproteins; IIEF‐5, 5‐International Index of Erectile Function; LDL, low density lipoproteins; LH, luteinizing hormone; ODI, Oxygen Desaturation Index; PRL, prolattin; SaO2, oxygen saturation.

**FIGURE 2:**
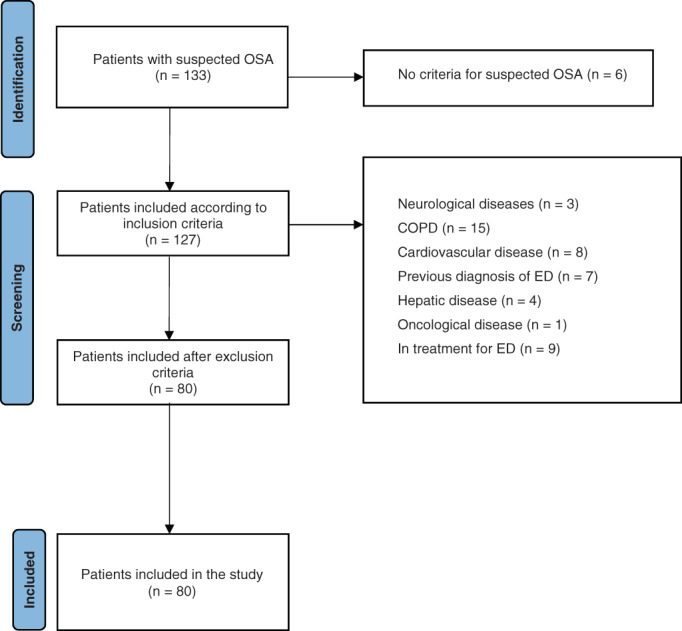
Scatter plots of IIEF‐5 and OSA parameters obtained via linear regression. AHI/h and IIEF‐5) *r* ^2^ = 0.218, *p* = 0.001; ESS and IIEF‐5) *r* ^2^ = 0.103, *p* = 0.026; ODI and IIEF‐5) *r* ^2^ = 0.200, *p* = 0.001; Sat02‐nadir (%) and IIEF‐5) *r* ^2^ = 0.218, *p* = 0.001; *r* ^2^ = 0.200, *p* = 0.001. ESS, Epworth Sleepiness Scale; ODI, Oxygen Desaturation Index; OSA, obstructive sleep apnoea

We found a statistically significant correlation between IIEF‐5 and age, hypertension, diabetes, ESS, AHI, ODI and SaO2‐nadir, reporting, in particular, an inverse correlation between OSA parameters and IIEF‐5 (Figure 2). No statistically significant differences were reported for VOTE patterns and IIEF‐5 score when ANOVA analysis was performed (V patterns, *p* = 0.397; O patterns, *p* = 0.413; T patterns, *p* = 0.433; E patterns, *p* = 0.322). Multiple linear regression analysis showed that age, diabetes, and SaO2‐nadir statistically significantly predicted IIEF‐5 [*F* (7.72) = 10.308, *p* < 0.0001, *r* ^2^ = 0.501] (Table 2).

**TABLE 2:** Univariate and multivariate linear regression between parameters and IIEF‐5

Univariate analysis			
	*r*	*r* ^2^	*P*
Age	−0.415	0.17	**<0.0001**
Hypertension	−0.327	0.11	**0.003**
Diabetes	−0.412	0.17	**<0.0001**
Smoking	0.147	0.02	0.194
BMI	−0.214	0.045	0.057
ESS	−0.375	0.14	**0.001**
AHI	−0.524	0.27	**<0.0001**
ODI	−0.516	0.26	**<0.0001**
SatO_2_‐nadir	0.569	0.32	**<0.0001**
Cholesterol	0.134	0.01	0.237
LDL	0.125	0.01	0.268
HDL	0.146	0.02	0.196
Triglycerides	−0.059	0.003	0.605
Albumin	−0.217	0.047	0.053
Testosterone	0.131	0.017	0.247
PRL	0.020	0.0004	0.860
FSH	−0.174	0.03	0.122
LH	−0.025	0.0006	0.825
PCR	0.176	0.031	0.119

Multivariate analysis				
	Beta coefficient	95% CI		*p*
Age	−0.164	−0.279	−0.05	**0.006**
Hypertension	−0.702	−2.928	1.524	0.532
Diabetes	−3.332	−6.373	−0.291	**0.032**
ESS	−0.210	−0.454	0.034	0.091
AHI	0.022	−0.14	0.184	0.789
ODI	−0.027	−0.225	0.170	0.784
SatO_2_‐nadir	0.558	0.149	0.967	**0.008**

Abbreviations: AHI, Apnea‐Hypopnea Index; BMI, body mass index; CRP, C‐reactive protein; ESS, Epworth Sleepiness Scale; FSH, follicle‐stimulating hormone; HDL, high density lipoproteins; IIEF‐5, 5‐International Index of Erectile Function; LDL, low density lipoproteins; LH, luteinizing hormone; ODI, Oxygen Desaturation Index; PRL, prolattin; SaO2, oxygen saturation.

Bold indicates statistically significant *P* < 0.05.

Among 48 ED patients, 45.8% reported mild ED (IIEF‐5 score: 17–21), 27.1% mild to moderate ED (IIEF‐5 score: 12–16), 20.8% moderate ED (IIEF‐5 score: 8–11) and 6.3% severe ED (IIEF‐5 score: 5–7). The main EES score was 9.13 ± 5.2 compared with 6.63 ± 3.24 in non‐ED patients [*t*(77.71) = −2.646; *p* = 0.010]. Similarly, the mean AHI score in ED subjects was 32.1 ± 16 whereas in non‐ED subjects was 24.5 ± 12.8 [*t*(77.9) = −3.767; *p* < 0.0001]; with analogous results for ODI [26.17 ± 11.8 vs. 17.43 ± 8.99 with *t*(76.5) = −3.754; *p* < 0.0001] and SaO2‐nadir (%) [84.38 ± 3.8 vs. 87.44 ± 3.19 *t*(73.9) = 3.749; *p* < 0.0001]. Age was also significantly different between ED and non‐ED patients with a mean 57.94 ± 8.96 versus 50.56 ± 7.75 respectively [*t*(78) = −3.801; *p* < 0.0001].

We found normal values of testosterone in OSA patients with (555.38 ± 150 ng/dl) and without ED (577.59 ± 135 ng/dl). Although lower values were reported in the first group, differences were not statistically significant.

Statistically significant differences were, instead, found in albumin concentration between the two groups [4.6 ± 0.62 in ED vs. 4.26 ± 0.57 in non‐ED, *t*(78) = −2.455, *p* = 0.016]. In addition, a higher prevalence of diabetes was, reasonably, found in ED patients [22.9% vs. 8.3% with *X* ^2^ (1) = 3.79, *p* = 0.015] (Table 3).

**TABLE 3:** Differences in parameters in patients with and without ED. Independent sample *t*‐test and Chi square test was performed for continuous and categorical variables respectively

	No ED		ED		*p*
	Mean	*SD*	Mean	*SD*	
Age	50.56	7.750	57.94	8.964	**<0.0001**
BMI	26.2253	2.67900	27.5615	3.61751	0.062
Pack/year	31.75	21.519	40.15	14.473	0.296
ESS	6.63	3.240	9.13	5.205	**0.010**
IIEF‐5	23.25	1.078	14.75	4.787	**<0.0001**
AHI/h	20.928	10.5065	32.106	16.0328	**<0.0001**
ODI	17.434	8.9935	26.179	11.7972	**<0.0001**
SaO_2_‐nadir (%)	87.44	3.192	84.38	3.813	**<0.0001**
Col	196.97	35.618	193.27	30.665	0.622
LDL	122.16	31.943	119.67	27.706	0.712
HDL	48.59	8.860	45.46	6.804	0.078
Triglycerides	128.25	35.360	140.79	38.592	0.145
Albumin (mg/dl)	4.263	0.5701	4.600	0.6230	**0.016**
Testosterone (ng/dl)	577.59	135.022	555.38	150.324	0.502
PRL (ng/ml)	11.8700	4.78751	11.5606	4.39693	0.769
FSH (mIU/ml)	4.759	2.9024	5.875	2.9980	0.103
LH (mIU/ml)	6.472	3.1089	6.610	3.1155	0.846
PCR (mg/L)	4.200	2.2606	3.413	2.3319	0.138
	Yes	No	Yes	No	
Hypertension	9 (28.1)	23 (71.9)	24 (50)	24 (50)	0.052
Diabetes	1 (8.3)	31 (96.9)	11 (22.9)	37 (77.1)	**0.015**
Smoking	8 (25)	24 (75)	13 (27.1)	35 (72.9)	0.836

Abbreviations: AHI, Apnea‐Hypopnea Index; BMI, body mass index; CRP, C‐reactive protein; ED, Erectile dysfunction; ESS, Epworth Sleepiness Scale; FSH, follicle‐stimulating hormone; HDL, high density lipoproteins; IIEF‐5, 5‐International Index of Erectile Function; LDL, low density lipoproteins; LH, luteinizing hormone; ODI, Oxygen Desaturation Index; PRL, prolattin; SaO2, oxygen saturation.

Bold indicates statistically significant *P* < 0.05.

## 4 DISCUSSION

OSA is a frequent medical condition, associated with sleep fragmentation, episodes of hypoxia and daytime somnolence (Kalejaiye et al., 2017; Zheng et al., 2020). Several cross‐sectional studies reported a prevalence of ED in patients with OSA ranging from 41% to 80%; interestingly, the OSA treatment with CPAP improves not only the ED but also the possible deficits in reproductive hormones (Hoyos et al., 2015; Zheng et al., 2020). Likewise, in our sample size, we found that 60% of OSA patients were diagnosed with ED with a mean IIEF‐5 value of 18.1 ± 5.6 (*SD*). Among possible causative factors linking ED and OSA, increased oxidative stress with reduced vasodilation and bioavailability of nitric oxide (NO), increased levels of catecholamines and endothelin, sleep fragmentation, reduced amounts of REM sleep, daytime sleepiness, impaired vigilance, prolonged bulbocavernosus reflex latency and decreased level of testosterone are considered the most important (Schulz et al., 2019). Furthermore, although literature data supported the hypothesis that OSA is associated with low libido and biochemical androgen deficiency, these relationships are not clearly assessed and are far less recognized (Pascual et al., 2018; Schulz et al., 2019).

In our study population, we found normal values of testosterone in the overall cohort and, similarly, both in patients with and without ED. This could be however related to the relatively young age of patients involved in our study. Hypoxia, reduced sleep time and sleep fragmentation can lead to the reduction of testosterone levels (Wittert, 2014). Moreover, as obesity is common among patients with OSA, BMI could be one of the most important determinants of testosterone levels in men with OSA (Barone et al., 2022; Shamim et al., 2015). This could be secondary to the increased expression of aromatase in adipose tissue and the reduction of sex hormone‐binding globulin (Colleluori et al., 2020). The relatively low BMI (27 ± 3.3) in our cohort of patients could probably explain the normal levels of testosterone and the lack of correlation between OSAS and hormonal status reported. It is however to be stated that the threshold of testosterone required to maintain an erection is relatively low and therefore, the impact of BMI and the expression of adipose‐tissue aromatase in erectile dysfunction could be significant only in men with severe cases of hypogonadism (Isidori et al., 2014). All enrolled subjects in our study complained of symptoms such as snoring, sleepiness, morning dry mouth and tiredness. The diagnosis of OSA was confirmed by overnight ambulatory RP. Our study population presented the commonest characteristics and comorbidities of apneic patients as: overweight, cigarette smoking, hypertension and diabetes mellitus type 2 (DM2; Bielicki et al., 2019). However, although we found in our patients a clear OSA symptomatology supported by diagnostic values of AHI (27.6 ± 15), the ESS score did not show values compatible with OSA (8.1 ± 4.6; Johns, 1991). These data stressed the importance to perform at least an overnight RP to define a clear diagnosis of OSA (Laratta et al., 2017).

In our cohort of 80 OSA patients, we found 26.3% of smokers, 41% of hypertensive, and 15% of type 2 diabetic subjects. So far, the relationship between OSA and smoking in the literature is inconclusive (Bielicki et al., 2019). Albeit some studies suggested that the AHI increases with the increase in the smoking rate, this relation was not further confirmed (Hsu et al., 2019). We found 26.3% of smokers in OSA patients, consistent with data reported in the literature which shows that 22% of newly diagnosed OSA patients were current/former smokers (Shao et al., 2020).

Regarding the relation between systemic hypertension and OSA, approximately half of our OSA patients suffered from systemic hypertension, in line with recent literature (Goldberger et al., 2008). In addition, we found 15% of patients suffering DM2 and overweight. The relationship between OSA, DM2 and obesity is probably multi‐directional. In particular, obesity and increased visceral fat are, in turn, perpetuating factors for DM2 in patients with OSA, causing leptin and insulin resistance (Berger & Polotsky, 2018; Jehan et al., 2018). In addition, OSA, in association with DM2, may affect glycemic control increasing the risk of DM2 complications (Khaire et al., 2020).

Among ED patients, we found a higher mean age and a higher prevalence of diabetes, confirming their role as risk factors for ED. The ESS score was higher than in non‐ED patients, although still below the cut‐off of 10. In addition, we found a significant correlation between ESS and RP parameters (AHI, ODI and SatO_2_‐nadir). In our study, we found a slightly higher level of albumin in OSA patients with ED. This data is in contrast with different studies reported in the literature that correlates hypoalbuminemia and ED in patients with chronic hepatitis and cirrhosis (Hunter et al., 2014). However, the causes of ED in patients with liver disease are not clear and the related disruption of vascular, hormonal and neurological integrity may result in an increased and independent sexual dysfunction risk (Kim et al., 2015). In addition, the altered ratio between free testosterone and albumin‐bound testosterone could explain ED in patients with hypoalbuminemia (Demir & Barlas, 2021). Due to these premises, our controversial data regarding albumin level could be an interesting point of discussion. However, the clinical significance of this finding requires further studies to evaluate the correlation with ED.

To the best of our knowledge, this is the first study correlating the ED parameters with DISE evaluation. Albeit the data regarding ED and DISE were inconclusive, our data demonstrated that ED correlated with OSA regardless of VOTE patterns, which are, however, prone to subjective evaluation. In particular, we further demonstrated how the severity of OSA influenced the erectile function, reporting negative correlations between OSA parameters (AHI, ESS and O_2_ Saturation Nadir) and IIEF‐5. In addition, to our univariate results and the mean differences in values among OSA patients with and without ED, multiple logistic regression analysis showed that age, diabetes and SaO_2_‐nadir were independent predictors of IIEF‐5 (*p* = 0.001, *p* = 0.001, *p* = 0.037 respectively) and consequently of ED. Our model showed a worsened IIEF‐5 score of −0.164 points for every year of age and −3.33 points for the presence of diabetes, whereas, for every 1% increase in SatO_2_‐nadir, IIEF‐5 score improved by 0.558 points. Despite the relatively small sample size, these data pointed out that the treatment and prevention of diabetes is essential to prevent ED, especially in patients with OSA, where diabetes itself represents an important comorbidity and a predictor of the ineffectiveness of phosphodiesterase‐5 inhibitors (PDE5‐I) therapy (Garrido‐Abad et al., 2021). In addition, the improvement of oxygenation with the treatment of OSA using CPAP or following oral appliances and/or surgery could, as well, improve ED.

We are conscious of several limitations which affect our study. First, the study design is prone to potential sampling bias; second, the RP and DISE evaluations, although made in the same clinic, were performed by different operators, without assessing the concordance among evaluations; third, the IIEF‐5 and the ESS assessment remain two subjective questionnaires that could have a wide intervariability; fourth, our sample size, although consistent with other studies reported in the literature, is still limited to evaluate the influence of DISE patterns in ED. Due to those limitations, the results obtained have to be, therefore, cautiously evaluated.

## 5 CONCLUSIONS

This study confirmed that OSA is a risk factor for ED. In particular, our data demonstrated that OSA parameters correlate with ED and age, while SatO2‐nadir and diabetes are independent predictors of ED. Our research pointed out that men presenting to the Ear, nose and throat (ENT) clinic with OSA are at significant risk of having ED. Therefore, males with OSA should be investigated for ED.

## AUTHOR CONTRIBUTIONS

Elena Cantone, Matteo Massanova and Felice Crocetto conceived and designed the study; Elena Cantone, Matteo Massanova, Fabrizio Corlianò, Fabio Esposito and Luigi Romano collected the data; Biagio Barone and Elena Cantone interpreted and analysed the data; Elena Cantone, Matteo Massanova and Biagio Barone wrote the manuscript; Davide Arcaniolo, Luigi Romano, Gaetano Motta, Elena Cantone, Biagio Barone and Antonio Celia revised the manuscript.

## CONFLICT OF INTEREST

The authors declare no conflict of interest.

## ETHICS STATEMENT

The present study protocol was reviewed and approved by the institutional review board of “Federico II” University of Naples (Reg No. 316/20). Informed consent was submitted by all subjects when they were enrolled.

## Data Availability

The data that support the findings of this study are available from the corresponding author upon reasonable request.
